# Stable fixation of an ultra-short femoral neck-preserving hip prosthesis: a 5-year RSA, DXA, and clinical prospective outcome study of 48 patients

**DOI:** 10.2340/17453674.2024.40074

**Published:** 2024-02-23

**Authors:** Janus D CHRISTIANSEN, Mogens LAURSEN, Gordon W BLUNN, Poul T NIELSEN

**Affiliations:** 1 Department of Orthopaedic Surgery and Orthopaedic Surgery Research Unit, Aalborg University, Aalborg, North Region; 2 Department of Clinical Medicine, Aalborg University, Aalborg, North Region, Denmark; 3 School of Pharmacy and Biomedical Sciences, University of Portsmouth, Portsmouth, UK

## Abstract

**Background and purpose:**

We previously showed promising primary stability and preservation of bone stock with the ultra-short neck-loading hip implant in total hip arthroplasty (THA). The aim of this study was to evaluate clinical outcome, implant stability, and bone mineral density (BMD).

**Methods:**

50 patients were treated with the ultra-short neck Primoris hip implant at baseline and 48 were available for evaluation at 5-year follow-up. 5 different patient-reported outcome measures (PROMs) including hip-specific scores, disease-specific and generic quality of life outcome measures, and an activity score were used. Furthermore, implant stability using radiostereometric analysis (RSA) and assessment of periprosthetic BMD using dual-energy X-ray absorptiometry (DXA) were applied.

**Results:**

By 1-year follow-up, all PROMs showed improvements and remained high at 5-year follow-up. After initial distal translation (subsidence) and negative rotation around the z-axis (varus tilt) the implant showed stable fixation at 5-year follow-up with no further migration beyond 12 months. In the regions of interest (ROI) 3 and 4, BMD remained stable. In ROI 2, further bone loss of 12% was found at 5-year follow-up.

**Conclusion:**

Clinical outcome including PROMs was satisfying throughout the 5-year follow-up period. The hip implant remains stable with both bone preservation and loss 5 years after surgery.

The long-term performance of cementless total hip arthroplasty (THA) is encouraging but still, non-physiological loading of the proximal femur is seen [1-3]. This may be a challenge in young and active patients who may lose bone in the long term. Primary inserted bone-sparing and more physiological bone-loading implants may give a better prognosis for subsequent revision where a standard stem could be an option. Results with bone-preserving implants in THA are ambiguous [4-8]. Hip implant designs are considered in different types, as some short stems resemble shorter versions of standard stems, while others are ultra-short without diaphyseal involvement as proposed by Khanuja et al. [9]. The meta-diaphyseal involvement in the femur of ultra-short stems makes them less comparable with the Primoris.

As a rough estimate, our ROI 1 and ROI 2 correspond to the Gruen zones 1 and 2 respectively while our ROI 4 corresponds to Gruen zones 6 and 7. As previously described, bone resorption in the proximal femur is seen after THA with different designs [1,3,10]. Albanese et al. showed a gain of 9% in ROI 2 but a loss of 7% in the calcar region with an ultra-short stem and a loss of 12% in ROI 2 and loss of 24% in the calcar region with a short stem [10]. Other studies showed substantial bone loss of more than 10% in ROI 2 and more than 25% in the calcar region [1-3].

Preclinical testing conducted by one co-author (GWB) and short-term evaluation of the Primoris hip implant have shown promising results [11]. The Primoris hip implant was introduced aiming for bone preservation and a more physiological loading pattern.

The aim of our study was to evaluate the 5-year performance of the Primoris hip implant regarding patient-reported outcome measures (PROMs), implant migration by RSA, and bone mineral density (BMD).

## Methods

The present study is a 5-year follow-up prospective cohort study [11].

### Patient selection

Patient recruitment for the present study occurred between July 2011 and February 2013. Males (18–65 years) and females (18–55 years) with end-stage osteoarthritis, non-compromised bone stock, and normal anatomy were eligible for inclusion. The 10-year lower age limit in females was due to the potential risk of osteoporosis and fracture after menopause. Detailed inclusion/exclusion criteria and patient characteristics for this highly selected group can be found in Table 1 (see Appendix). 50 hips in 5 females and 45 males (30 right and 20 left) were treated with the Primoris implant. The mean age was 52 years (25–65) and the body mass index (BMI) was 29 (range 22–37).

**Table 1:** Exclusion criteria

Patients who did not understand the given information
Competing disorders requiring treatment with anti-inflammatory drugs
Estimated remaining life expectancy of less than 10 years
Rheumatoid arthritis or other types of arthritis
Previous surgery on relevant hip
Pain normative and disabling osteoarthritis of the ipsilateral knee
Comorbidity (ASA group 3–5)
Neurological disorder compromising the motor skills and rehabilitation courses
Pregnancy
Osteoarthritis secondary to Calvé–Legg–Perthes’ disease and juvenile epiphysiolysis coxae
Acetabular dysplasia and secondary subluxation (Crowie grade II–IV)
Previously established osteoporosis or osteoporosis detected by DXA scan prior to surgery
Ongoing treatment with osteoporosis medications (i.e., calcium and vitamin D, bisphosphonates etc.)
Aseptic necrosis of the femoral head (post-traumatic, idiopathic)
Deformity of the femoral neck (femoral length, measured medially ≤ 15 mm)
Varus or valgus deformity in the proximal femur including femoral collum angle < 125° or > 145°
Femoral retroversion or anteversion

### Surgery and implant

**Figure 1:**
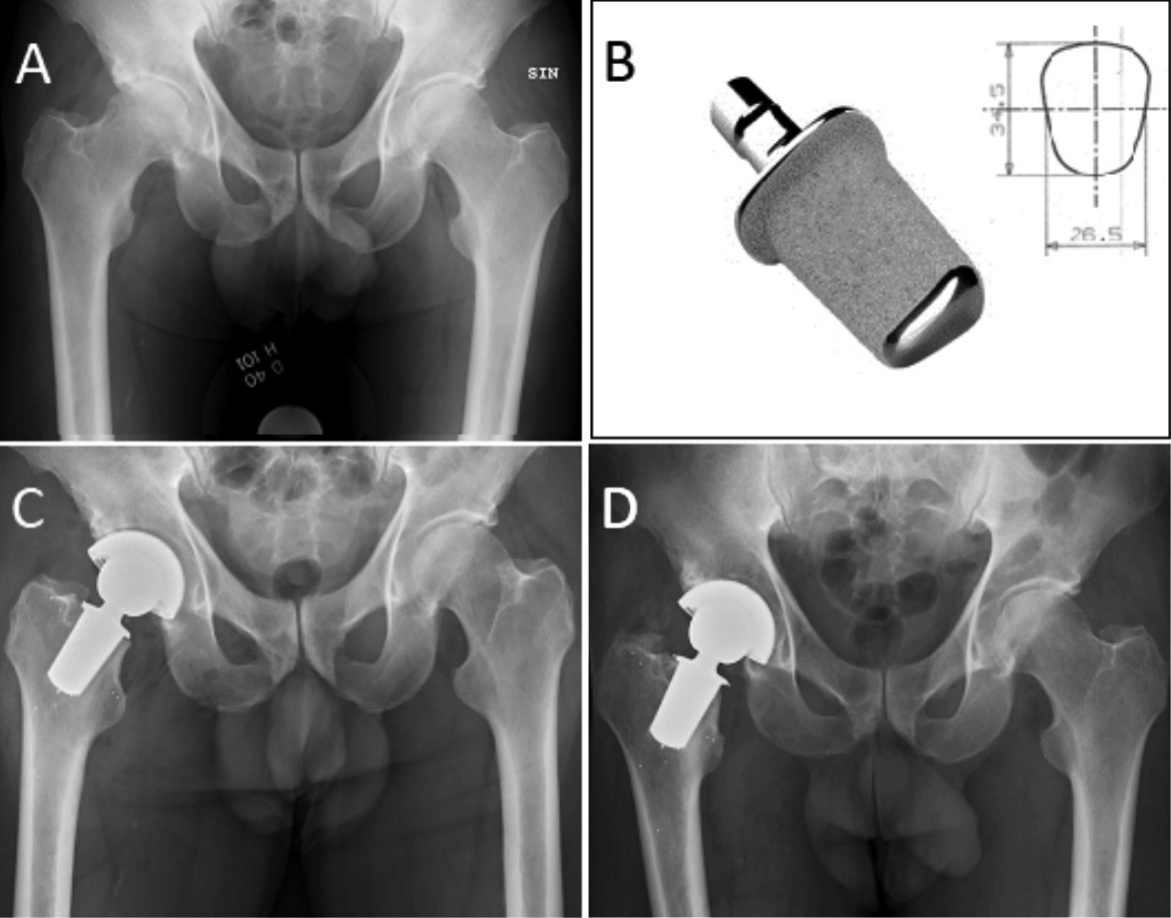
Radiographic imaging of 1 patient in the present study. A. Preoperative radiograph with end stage osteoarthritis of the right hip joint. B. Picture of the Primoris including a drawing of the cross-sectional geometry of the implant. C. Radiograph of the same patient with the Primoris in situ with tantalum beads in the proximal femur and markers attached to the implant at day 1. D. 5-year follow-up.

Surgery was performed by one surgeon (PTN) using the posterior approach. For future RSA analysis, tantalum beads were inserted in the proximal femur. The implant was also marked with 3 tantalum beads (Figure 1). To mitigate the risk of failure, partial weightbearing using crutches was allowed in the first 6 weeks postoperatively as a precaution, as this implant has no stem to augment initial stability. The uncemented Primoris implant (Biomet, Warsaw, IN, USA, today Zimmer Biomet) with a titanium alloy and hydroxyapatite coating (BoneMaster) was inserted (Figure 1). A Regenerex cup (Biomet) with an E-poly liner (Biomet) and CoCr femoral head (32 or 36 mm) was used. This implant differs from traditional implants by its short stem, fixed primarily in the remaining metaphyseal bone of the neck region, not involving the diaphysis and with a cross-sectional implant geometry designed to resist torsional forces (Figure 1). The initial stability is enhanced by a press-fit insertion in compacted neck and metaphyseal bone. Details on implant design and surgical procedure have been described previously [11].

### Patient-reported outcome measures

We used the hip-specific Harris Hip Score (HHS) and Oxford Hip Score (OHS), the disease-specific quality-of-life measure Western Ontario & McMaster Universities Arthritis Index (WOMAC), the generic quality-of-life measure EuroQol 5-dimension health-related quality of life measure (EQ-5D3L), and the activity score from the University of California Los Angeles (UCLA) as PROMS. Best outcome scores are 100 points for HHS and 48 points for OHS. WOMAC has a raw-score range of 0–96. This raw score is multiplied by 100/96 giving a reported score of 0 (worst) to 100 (best). EQ5D3L contains 5 dimensions with 3 levels for response with a score from 0 as worst to 1 as the best possible health state. The Danish value set was used to calculate index values. UCLA activity score determines the patients’ activity level through a graduation of questions, with 10 as best outcome. PROMs were filled in at the outpatient clinic prior to consultation with the surgeon and evaluated preoperatively, and at 6 weeks, 6 months, 1 year, 2 years, and 5 years postoperatively.

### Evaluation of migration and bone preservation

Implant migration pattern and BMD were evaluated by RSA and dual-energy X-ray absorptiometry (DXA) scans respectively at day 1 (baseline), 6 weeks, 6 months, 1 year (including double measurements), 2 years, and 5 years postoperatively. RSA was analyzed using model-based RSA (MBRSA) software (RSACore, Leiden University, the Netherlands) as described in Christiansen et al. [11]. Translational and rotational migrations were determined relative to the x-, y-, and z-axis and maximum total point of motion (MTPM) was calculated for each patient. RSA assessment was performed in accordance with Valstar et al. [12]. Baseline values for migration where set to zero movement with the first RSA imaging at day 1 after operation. In accordance with a previously described region of interest (ROI) protocol, 4 ROIs were analyzed [13]. The Norland XR-36 scanner was used, and scan reports showed a coefficient of variation of 0.43 to 0.74.

### Statistics

PROM results were reported as descriptive statistics including median and interquartile range (IQR). Migration was presented as median and interquartile range (IQR) (Table 2). The Wilcoxon signed-rank test was used for comparison of RSA translations and rotations as the values were not normally distributed. P values less than 0.05 were considered significant. Data for BMD was normally distributed and differences in BMD at each ROI were calculated using a paired t-test (Table 3). Box and whisker plots were performed for all outcomes to identify outliers. In our statistical package individuals with any missing data were explicitly excluded from the analysis. Regarding missing data (RSA/migration and DXA/BMD) we analyzed repeated measurements using a multiple imputation technique to ensure that our available case could be used as the default approach. The Appendix represents data from these approaches comparable with Table 2 and Table 3. STATA MP 16.0 (StataCorp LLC, College Station, Texas, USA) was used for statistical analysis.

**Table 2:** Median values (interquartile range) of implant migration of the Primoris implant (n = 43)

Migration	6 weeks	6 months	12 months	24 months	60 months	P value ^a^ 0 vs. 6 weeks	P value ^a^ 6 weeks vs. 2 years	P value ^a^ 6 weeks vs. 5 years
Translation, mm								
X	0.08 (–0.02 to 0.22)	0.07 (–0.04 to 0.27)	0.09 (–0.05 to 0.29)	0.10 (0.03 to 0.39)	0.08 (–0.06 to 0.30)	0.01	0.02	0.7
Y	–0.06 (–0.49 to –0.01)	–0.04 (–0.28 to 0.07)	–0.03 (–0.30 to 0.08)	–0.03 (–0.36 to 0.03)	–0.03 (–0.43 to 0.09)	0.001	0.005	0.001
Z	0.02 (–0.17 to 0.24)	0.00 (–0.18 to 0.26)	–0.01 (–0.14 to 0.24)	–0.01 (–0.16 to 0.18)	0.01 (–0.18 to 0.29)	0.6	0.6	0.9
Rotation, °								
X	–0.06 (–0.30 to 0.10)	–0.09 (–0.39 to 0.26)	–0.04 (–0.36 to 0.28)	–0.01 (–0.45 to 0.27)	–0.02 (–0.46 to 0.37)	0.2	0.3	0.6
Y	0.02 (–0.27 to 0.33)	0.18 (–0.25 to 0.40)	0.10 (–0.12 to 0.44)	0.14 (–0.10 to 0.44)	0.08 (–0.18 to 0.45)	0.6	0.2	0.2
Z	–0.27 (–1.05 to –0.09)	–0.54 (–1.03 to –0.06)	–0.39 (–0.91 to –0.06)	–0.40 (–0.95 to –0.14)	–0.32 (–1.04 to –0.09)	0.001	0.02	0.3

a Wilcoxon signed-rank test.

**Table 3:** Mean values of bone mineral density (g/cm^2^) including (SD) measured at Day 1, and 2 and 5 years after surgery in the 4 regions of interest (ROI) including the mean differences between day 1 and 5 years and between 2 years and 5 years with 95% confidence interval (CI) and P values

ROI	day 1	2 years	5 years	Day 1 vs. 5 years		2 years vs. 5 years	
				Mean difference (CI)	P value^a^	Mean difference (CI)	P value^a^
1	0.93 (0.13)	0.88 (0.14)	0.89 (0.14)	–0.04 (–0.07 to –0.01)	0.008	0.01 (–0.01 to 0.03)	0.06
2	1.37 (0.18)	1.25 (0.20)	1.21 (0.19)	–0.16 (–0.19 to –0.12)	0.001	–0.04 (–0.06 to –0.01)	0.008
3	1.87 (0.13)	1.89 (0.20)	1.88 (0.37)	0.01 (–0.03 to 0.05)	0.5	–0.01 (–0.06 to 0.04)	0.9
4	1.40 (0.15)	1.45 (0.21)	1.41 (0.15)	0.02 (–0.02 to 0.05)	0.2	–0.03 (–0.07 to 0.01)	0.1

a Paired sample t-test.

### Ethics, registration, funding, and disclosures

Written and informed consent was obtained from all patients, who were enrolled according to the guidelines for observational studies in epidemiology (STROBE) [14] and the Helsinki Declaration. The local ethics committee approved the study on February 2, 2011 (approval no. N-20100054). The study is also registered at ClinicalTrials.gov (NCT01326832). This study was co-financed by Aalborg University Hospital and Biomet Europe. Biomet Europe has manufactured the Primoris hip implant, co-financed the RSA analysis, and covered the difference in expenses between this new implant and the standard implant used in our clinic. The Primoris implant is patented (CE560346) and licensed to Zimmer Biomet Inc. None of the authors received any personal compensation from Biomet Europe. Complete disclosure of interest forms according to ICMJE are available on the article page, doi: 10.2340/17453674.2024.40074

## Results

**Figure 2:**
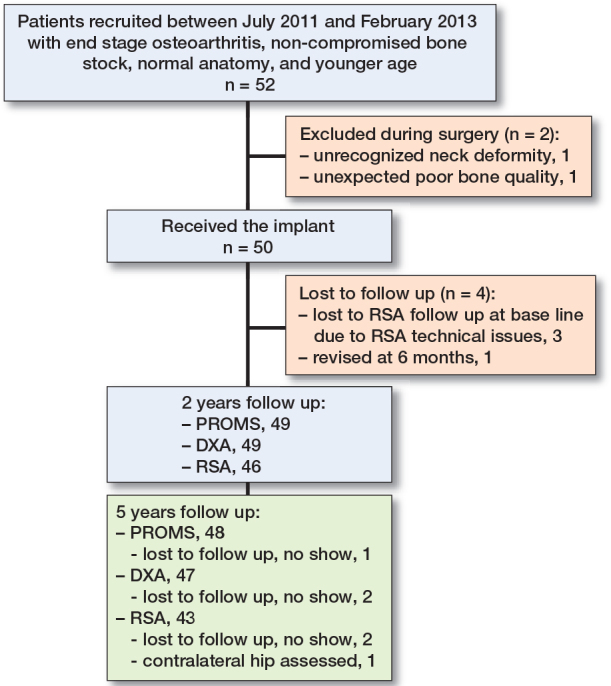
Flowchart of the study.

1 patient was revised with a traditional stem by 6-month follow up (FU) due to very large migrations (6-month MTPM was 18 mm and subsidence 12 mm) (Figure 2). Due to loss or lack of visible markers, 3 patients were excluded from future migration analysis as previously described [11] (Figure 2). 1 patient did not show up at 5 years FU (Figure 2). 1 patient missed the RSA assessment at 5-year FU due to unexplained reasons while another patient had RSA assessment performed on the contralateral hip, which is not included in the study, leaving 43 patients for RSA assessments (Figure 2). 1 patient did not show up for DXA at 5 years (Figure 2).

### Patient-reported outcome measures

**Figure 3:**
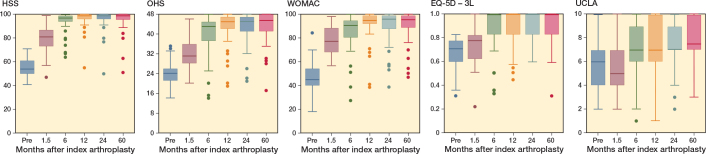
Box plots showing the clinical scores at each FU interval. Boxes are interquartile range (IQR) with horizontal line indicating medians. The whiskers represent min and max values within 1.5 times the IQR. The closed circles are outliers that are > 1.5 times the IQR. HHS = Harris Hip Score, OHS = Oxford Hip Score, WOMAC = Western Ontario & McMaster Universities Arthritis Index, EQ-5D = EuroQol 5-dimension health-related quality of life measure, and UCLA = University of California Los Angeles.

By 1-year FU great improvements were seen in HHS, OHS, and WOMAC compared with preoperative scores (Figure 3). All 3 scores remained high at 5-year FU (Figure 3). The EQ5D3L and UCLA activity score showed improvement at 6 months FU. In each PROM score there were 3–5 outliers with very low scores during the 5-year FU. Further improvement or degradation was not found (Figure 3).

### RSA

**Figure 4:**
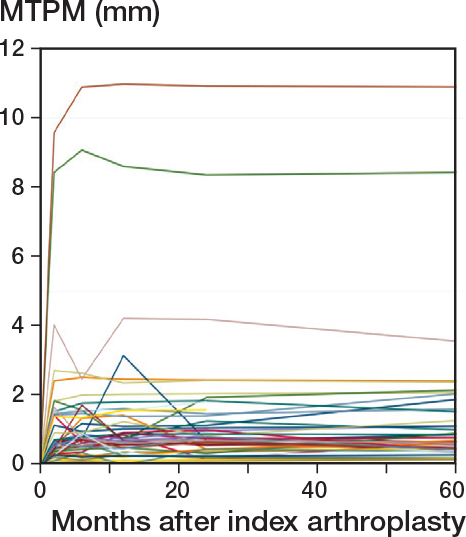
Individual maximal total point motion (MTPM) during 60 months of follow-up.

Most implant migration occurred within the first 6 weeks before settlement and demonstrated stable fixation throughout the rest of the period (Figure 4). Distal translation (subsidence) was significant between day 1 and 6 weeks (P = 0.001) and proximal translation (lift-off) at 24 months and 60 months compared with 6 weeks (P = 0.005 and 0.001, respectively) (Table 2). Migration along the x- and z-axes (medial/lateral and anterior/posterior migration) remained stable after initial settlement with no significant difference throughout the follow-up period (Table 2). A significant negative rotation around the z-axis (abduction/varus tilt) was found between day 1 and 6 weeks (P = 0.001) and between 6 weeks and 24 months (P = 0.02). A continuous varus tilt remained throughout the follow-up period.

### BMD

**Figure 5:**
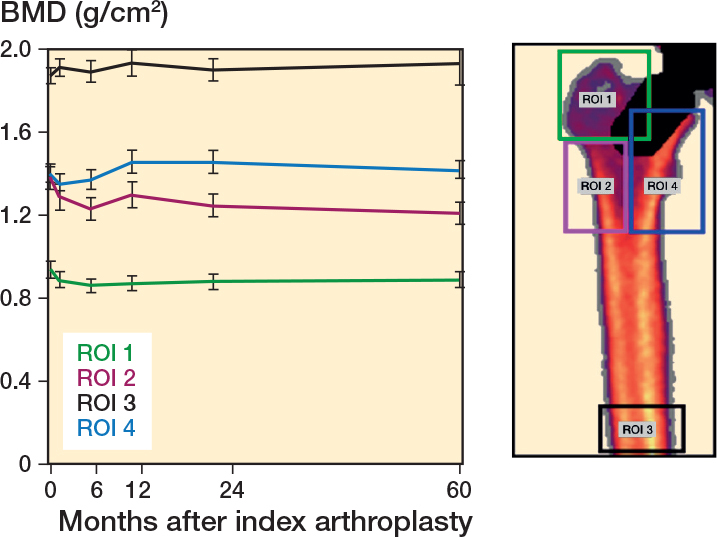
Changes in bone mineral density. (BMD, g/cm^2^) in the 4 ROIs over 60 months including DXA image of the Primoris in the proximal part of the femur marked with the 4 regions of interest (ROI 1–4). Error bars represent the standard error of the mean. ROI 1 = greater trochanter (green line), ROI 2 = lateral (purple line), ROI 3 = diaphysis (black line), and ROI 4 = lesser trochanter and calcar (blue line).

BMD remains stable after 5 years in ROI 1, ROI 3, and ROI 4 (Figure 5). In ROI 2, significant bone losses with differences of –11.6% (CI –14.2 to –8.9) at 5 years compared with day 1 and –2.6% (CI –4.5 to –0.7) at 5-year compared with 2-year FU were found (Table 3).

### Outliers, lost to follow-up, and missing data

Only 1 patient has multiple outlier positions over time across PROMs and RSA (Table 4, see Appendix and Figure 3). In general, common features were limited, as most patients were either outliers in the PROM section or RSA section. A few outliers were found in ROI 2 to ROI 4 when considering a position lower than minimum values. ROI 1 did not have any outliers and is thus not included in Table 4 (see Appendix). The 2-year data for patients lost to FU at 5 years is presented in Table 5 (see Appendix). This table also includes the 3 patients not participating in any RSA assessments due to technical issues with lack of markers. 2 of these 3 patients are outliers as seen in Table 4 (see Appendix) regarding PROMs. The 5 other patients missing at random at 5 years were not outliers at 2-year FU according to the data (Table 5, see Appendix). As seen in the Appendix, our available cases resemble the results from repeated measurements of missing data concerning both BMD and RSA including RSA results expressed as mean (CI) for the available cases (Tables 6–9, see Appendix).

**Table 4:** Overview of outliers. Number indicates how many times a patient has been an outlier in a specific outcome across the different FU intervals throughout the 5 years

	HHS	OHS	WOMAC	EQ-5D	UCLA	ROI2	ROI3	ROI4	Translation			Rotation		
									X	Y	Z	X	Y	Z
P1				1									1	
P3								1						
P6					1									
P8	1													
P12									5	5	5	5	4	5
P13	4	2	3		2							2	1	
P14	1	2	2	2										
P16	2	2	3	1	1				N/A	N/A	N/A	N/A	N/A	N/A
P17					1									
P18								2						
P22	4	4	4	2					4	1		1	4	5
P23	2													
P25		1	1								1		1	
P26						1								
P27			1						1					1
P30									2					
P31							1		5			1		5
P32									5	5	3	3	3	5
P33					1							1		
P34							1							
P36													3	
P37	5	2	4	1					N/A	N/A	N/A	N/A	N/A	N/A
P41									1					
P42													1	
P43	1	2	2											
P45									1		1			
P50	1	1	1	1										
P51			1											

HHS = Harris Hip Score, OHS = Oxford Hip Score, WOMAC = Western Ontario & McMaster Universities Arthritis Index, EQ-5D = EuroQol 5-dimension health-related quality of life measure, UCLA = University of California Los Angeles. ROI = region of interest. N/A = not available.

**Table 5:** Values at 2-year FU of patients lost to 5-year FU in either or all outcomes

	Data from 2-year FU										
	HHS	OHS	WOMAC	EQ-5D	UCLA	ROI1	ROI2	ROI3	ROI4	Y-trans	Z-rot
P5 (missed 5-year DXA)	100	47	98	1.00	7	0.89	1.01	2.18	1.37	–0.01	–0.75
P7 (missed 5-year FU)	99	47	100	1.00	5	0.99	1.38	1.90	1.75	–0.57	–1.60
P16 (missed all RSA)	77	27	57	0.69	3	0.74	1.18	1.76	1.42	N/A	N/A
P37 (missed all RSA)	80	32	53	0.69	4	0.91	1.29	1.54	1.14	N/A	N/A
P39 (missed 5-year RSA)	100	48	100	1.00	7	0.86	1.16	2.01	1.42	–0.68	–1.60
P46 (missed all RSA)	98	44	93	0.83	5	0.91	1.68	2.07	1.46	N/A	N/A
P48 (missed 5-year RSA)	97	45	97	1.00	7	0.78	1.01	1.46	1.17	–0.09	–0.23

HHS = Harris Hip Score, OHS = Oxford Hip Score, WOMAC = Western Ontario & McMaster Universities Arthritis Index, EQ-5D = EuroQol 5-dimension health-related quality of life measure, UCLA = University of California Los Angeles. ROI = region of interest, DXA = dual-energy X-ray absorptiometry, RSA = radiostereometric analysis.

**Table 6:** Repeated measurements including multiple imputation analysis of the mean values of bone mineral density (g/cm^2^) including (SD) measured at Day 1, and 2 and 5 years after surgery in the 4 regions of interest (ROI) (n = 49) with the mean differences between Day 1 and 5 years and between 2 years and 5 years with 95% confidence interval (CI)

ROI	day 1	2 years	5 years	Day 1 vs. 5 years		2 years vs. 5 years	
				Mean difference (CI)	P value^a^	Mean difference (CI)	P value^a^
1	0.94 (0.12)	0.88 (0.13)	0.89 (0.14)	–0.04 (–0.07 to –0.01)	0.008	0.02 (0.01 to 0.03)	0.03
2	1.37 (0.18)	1.25 (0.20)	1.21 (0.19)	–0.16 (–0.20 to –0.13)	0.001	–0.04 (–0.06 to –0.02)	0.003
3	1.88 (0.14)	1.89 (0.20)	1.89 (0.18)	0.01 (–0.30 to 0.05)	0.6	–0.01 (–0.06 to 0.04)	1.0
4	1.40 (0.15)	1.45 (0.20)	1.42 (0.15)	0.01 (–0.02 to 0.05)	0.3	–0.03 (–0.07 to 0.01)	0.1

a Paired sample t-test.

**Table 7:** Repeated measurements including multiple imputation expressed as median values (IQR) of the migration of the Primoris implant

Migration	6 weeks (n = 46)	6 months (n = 46)	12 months (n = 46)	24 months (n = 46)	60 months (n = 43)	P value ^a^ 0 vs.6 weeks	P value ^a^ 6 weeks vs. 2 years	P value ^a^ 6 weeks vs. 5 years
Translation, mm								
X	0.09 (–0.02 to 0.31)	0.09 (–0.02 to 0.28)	0.09 (–0.04 to 0.32)	0.11 (0.03 to 0.39)	0.08 (–0.05 to 0.33)	0.003	0.02	0.7
Y	–0.07 (–0.54 to –0.01)	–0.05 (–0.47 to 0.07)	–0.03 (–0.49 to 0.07)	–0.04 (–0.42 to 0.03)	–0.03 (–0.46 to 0.08)	0.001	0.007	0.001
Z	0.01 (–0.17 to 0.24)	–0.01 (–0.18 to 0.02)	–0.01 (–0.15 to 0.24)	–0.01 (–0.16 to 0.18)	0.01 (–0.19 to 0.29)	0.6	0.7	0.9
Rotation, °								
X	–0.06 (–0.30 to 0.12)	–0.08 (–0.39 to 0.31)	–0.06 (–0.36 to 0.28)	–0.01 (–0.45 to 0.27)	–0.04 (–0.46 to 0.37)	0.2	0.3	0.7
Y	0.07 (–0.25 to 0.40)	0.18 (–0.24 to 0.50)	0.14 (–0.12 to 0.50)	0.16 (–0.10 to 0.45)	0.17 (–0.14 to 0.47)	0.3	0.3	0.2
Z	–0.29 (–1.10 to –0.09)	–0.54 (–1.13 to –0.07)	–0.40 (–0.97 to –0.07)	–0.44 (–1.02 to –0.14)	–0.32 (–1.08 to –0.10)	0.001	0.02	0.3

a Wilcoxon signed-rank test.

**Table 8:** Implant migration expressed as mean (SD) translation and rotation of the Primoris hip implant (n = 43) with the mean differences between 6 weeks and 2 years and between 6 weeks and 5 years with 95% confidence interval (CI)

	6 weeks	2 years	5 years	6 weeks vs. 5 years		2 years vs. 5 years	
				Mean difference (CI)	P value^a^	Mean difference (CI)	P value^a^
Translation, mm							
X	0.16 (0.54)	0.24 (0.52)	0.19 (0.54)	–0.08 (–0.15 to –0.01)	0.03	–0.03 (–0.09 to 0.04)	0.4
Y	–0.38 (0.84)	–0.33 (0.91)	–0.31 (0.91)	–0.05 (–0.09 to –0.01)	0.01	–0.07 (–0.11 to –0.03)	0.001
Z	0.03 (0.46)	–0.01 (0.35)	–0.00 (–0.35)	0.04 (–0.05 to 0.13)	0.4	0.03 (–0.07 to 0.13)	0.6
Rotation, °							
X	–0.13 (0.85)	–0.09 (0.65)	–0.12 (0.70)	–0.03 (–0.21 to 0.14)	0.7	–0.01 (–0.18 to 0.15)	0.9
Y	0.08 (0.82)	0.14 (0.58)	0.16 (0.76)	–0.06 (–0.27 to 0.14)	0.5	–0.08 (–0.30 to 0.14)	0.5
Z	–0.87 (1.84)	–0.98 (2.06)	–0.95 (2.05)	0.10 (–0.01 to 0.21)	0.06	0.08 (–0.03 to 0.18)	0.2

a Wilcoxon signed–rank test.

**Table 9:** Repeated measurements including multiple imputation analysis of the mean values of implant migration expressed as mean (SD) translation and rotation of the Primoris hip implant (n = 46) with the mean differences between 6 weeks and 2 years and between 6 weeks and 5 years with 95% confidence interval (CI)

	6 weeks	2 years	5 years	6 weeks vs. 5 years		2 years vs. 5 years	
				Mean difference (CI)	P value^a^	Mean difference (CI)	P value^a^
Translation, mm							
X	0.17 (0.53)	0.25 (0.51)	0.20 (0.52)	–0.07 (–0.14 to –0.01)	0.04	–0.03 (–0.09 to 0.03)	0.4
Y	–0.38 (0.82)	–0.33 (0.88)	–0.31 (0.88)	–0.04 (–0.08 to –0.01)	0.01	–0.07 (–0.10 to –0.03)	0.001
Z	0.03 (0.45)	–0.00 (0.35)	–0.00 (0.35)	0.03 (–0.05 to 0.12)	0.5	0.03 (–0.06 to 0.12)	0.6
Rotation, °							
X	–0.12 (0.83)	–0.09 (0.64)	–0.11 (0.69)	–0.37 (–0.61 to –0.12)	0.004	–0.32 (–0.58 to –0.07)	0.01
Y	0.10 (0.80)	0.17 (0.57)	0.18 (0.74)	–0.05 (–0.25 to 0.13)	0.6	–0.07 (–0.28 to 0.13)	0.5
Z	–0.89 (1.79)	–0.98 (2.00)	–0.96 (1.99)	0.09 (–0.01 to 0.20)	0.07	0.07 (–0.02 to 0.17)	0.1

a Wilcoxon signed–rank test.

## Discussion

In this study we evaluated the 5-year performance of an ultra-short-neck anchored hip implant with regards to PROMs, BMD, and RSA. We showed stable implant fixation and satisfying clinical results with the Primoris. No adverse events regarding dislocation of the hip joint or periprosthetic infections were found.

### Clinical outcome

All PROMs improved and stabilized at 1-year FU with satisfying results. These results are in line with those published in other studies including both traditional and short stems concerning clinical outcome after hip arthroplasty, despite the fact that populations in the different studies are not comparable exactly [15-18]. Many studies have only 1 or 2 different PROMs. In the present study we included a wide range of validated PROMs. Thus, disease-specific outcome measures and other measuring tools in functioning, pain, health-related quality of life, mental health, and activity level were utilized. Some patient factors such as comorbidities, age, and health are not accounted for in HHS and OHS, which could compromise the specific outcome. Therefore, an adequate variety or combination of different outcome measures is preferable [19]. However, the different scores used in the present study seemed to follow the same improvement pattern (Figure 3). In general, outliers from the PROMs data were not identical to outliers from the migration analysis (Table 4, see Appendix). 5 patients did not have successful PROM results. However, this could not be explained by RSA or radiological findings.

### Migration

A weightbearing regime could potentially be a disadvantage with compromised fixation. Other studies have shown early weightbearing is not inferior to weightbearing regimes [18,20]. Most migration occurred during the first 6 months with major migrations within the first 6 weeks, as previously described [11]. Subsidence and varus tilt were the most important migration patterns. Such migration patterns could lead to implant failure with aseptic loosening due to stress-shielding. This could explain the reduced BMD in ROI 2, due to non-physiological loading. Future FU of the present study will be performed to uncover any development of this potential problem. No further migration was detected throughout the 5-year FU, consistent with other studies [1,21].

### Bone remodeling

A continuous positive bone turnover in ROI 3–4 is still present at 5 years and higher than day 1, whereas the bone loss in ROI 1 has stabilized from 2- to 5-year FU (Table 3). The proximal femoral bone is still preserved at 5 years after index surgery. These results of BMD are in accordance with other studies on ultra-short implants [21,22]. After initial changes in BMD during the first 12 months, a steady decline or plateauing was found in ROI 2 and ROI 4. These findings are in line with other studies where bone remodeling decreases after 1 year, resembling that of normal aging [3,6]. The preservation of bone along the calcar area in ROI 4 after 5 years is encouraging, with a 1.8% gain when compared with day 1 after surgery (Table 3). Concerning ROI 2, bone loss of 11.6% is considerable. However, bone loss of nearly 9% was already found at 2-year FU [11]. Thus, a slower decline was seen in the following 3 years, which reflects the loading pattern in the proximal femur with more compressional forces medially and more tensional forces laterally [23]. Future assessment using DXA analysis is needed to reveal any further decline or plateauing in this region of concern.

### Strengths

The Primoris implant was introduced according to the paradigm of stepwise introduction of new implants [24]. As initial preclinical testing was promising, this was followed by the present pilot study representing clinical step I in the stepwise introduction. Outcomes usually are more favorable for designers than for non-designer surgeons. However, this clinical step I study was supported by RSA and DXA assessment, which are very strong objective measures. RSA is one of the most accurate techniques to measure implant migration and identify cases of aseptic implant loosening.

### Limitations

The patients are highly selected young patients not deviating from normal anatomy and with good bone stock, therefore all clinical and paraclinical outcomes should be interpreted with caution. Thus, results in this study are not considered applicable for reproducibility in a general population. A randomized controlled trial has been established and is still ongoing and represents clinical step II in the stepwise introduction of new implant designs. Our statistical package explicitly excluded all individuals with any missing value in any outcome. To ensure that our available cases could be used as the default approach, we analyzed repeated measurements using a multiple imputation technique. The Appendix represents data from this approach comparable to Table 2 (RSA) and Table 3 (BMD). Concerning both BMD and RSA it is our impression that the impact of missing data in these analyses is limited (Tables 6–9, see Appendix). Thus, these results did not change the conclusions as data was not biased by missing data.

### Conclusions

Clinical outcome including PROMs was satisfying throughout the 5-year FU. The hip implant remains stable with both bone preservation and loss 5 years after surgery.

In perspective, longer FU is needed to assess the durability of this implant and to discover whether revision of this stem is more favorable than revision of conventional stems.

## Notes

JDC: formal analysis including statistical analysis, investigation, methodology, project administration, writing original draft. ML: formal analysis, project administration, supervision, validation, writing, review, and editing. GWB: writing, review, and editing. PTN: conceptualization, methodology, project administration, supervision, writing, review, and editing.

The authors thank their research nurses Gitte B Hansen, Ulla Hornum, and Merete Hesselund for organizing appointments and follow-up for the patients and other assistance, and research assistant Heidi L Poulsen for RSA analysis, assistance, and support.

Handling co-editors: Keijo Mäkelä and Robin Christensen

*Acta* thanks Harald Brismar and Georgios Tsikandylakis for help with peer review of this manuscript.
